# Giovanni Antonio Scopoli's *De Hydrargyro Idriensi Tentamina* (1761): Mercury Mining, Mercurialism, and Preventive Reasoning in Eighteenth‐Century Occupational Medicine

**DOI:** 10.1002/ajim.70128

**Published:** 2026-08-23

**Authors:** Alberto Zanatta, Paola Mason, Sofia Bollini, Giovanni Magno

**Affiliations:** ^1^ Department of Cardiac, Thoracic, Vascular Sciences and Public Health University of Padua Italy; ^2^ Università della Svizzera Italiana; Università degli Studi della Campania Luigi Vanvitelli, Napoli, Italy; ^3^ Morgagni Museum of Human Anatomy—University Museums Centre CAM, University of Padua Italy

**Keywords:** history of occupational health, industrial toxicology, mercurialism, mercury miners, mercury poisoning, occupational medicine, Ramazzini, Scopoli, Thackrah

## Abstract

Giovanni Antonio Scopoli (1723–1788), a physician‐naturalist of the Enlightenment, is primarily remembered for his contributions to botany and entomology. Less attention has been paid to his medical work *De hydrargyro Idriensi Tentamina physico‐chymico‐medica* (1761), written during his fifteen years of service as physician at the mercury mines of Idrija (present‐day Slovenia). Its third section, *De morbis fossorum hydrargyri*, offers a detailed occupational‐medical account of disorders and diseases among mercury miners and smelter workers, including tremor, salivation, stomatitis, respiratory disorders, and other conditions associated with mining labor. This article reassesses Scopoli's work within the history of occupational medicine, comparing his site‐specific approach with the broader nosology of Bernardino Ramazzini and the later reform‐oriented tradition associated with 19th‐century industrial medicine. Rather than presenting Scopoli as a neglected founder of the discipline, we argue that the *Tentamina* represents a distinctive 18th‐century form of occupational‐medical inquiry: a monographic, workplace‐based, clinically grounded study that linked toxic exposure, working conditions, and prevention before the emergence of modern industrial toxicology. The work remains substantially unknown in Anglophone historiography and deserves wider recognition as a significant example of pre‐industrial occupational‐medical inquiry.

## 1 Introduction

Histories of occupational medicine have often been organized around a small number of canonical texts. Bernardino Ramazzini's *De morbis artificum diatriba* (1700) remains the most influential early synthesis of diseases associated with work exposures, while Charles Turner Thackrah's *The Effects of Arts, Trades, and Professions on Health and Longevity* (1831) has commonly been read as a key text of early industrial occupational medicine. Between these two landmarks, however, a significant body of work was produced by physicians who, stationed at specific industrial sites, developed detailed monographic studies of occupational disease based on direct clinical experience. Among these, the contribution of Giovanni Antonio Scopoli (1723–1788) deserves particular attention (Figure 1).

**Figure 1:**
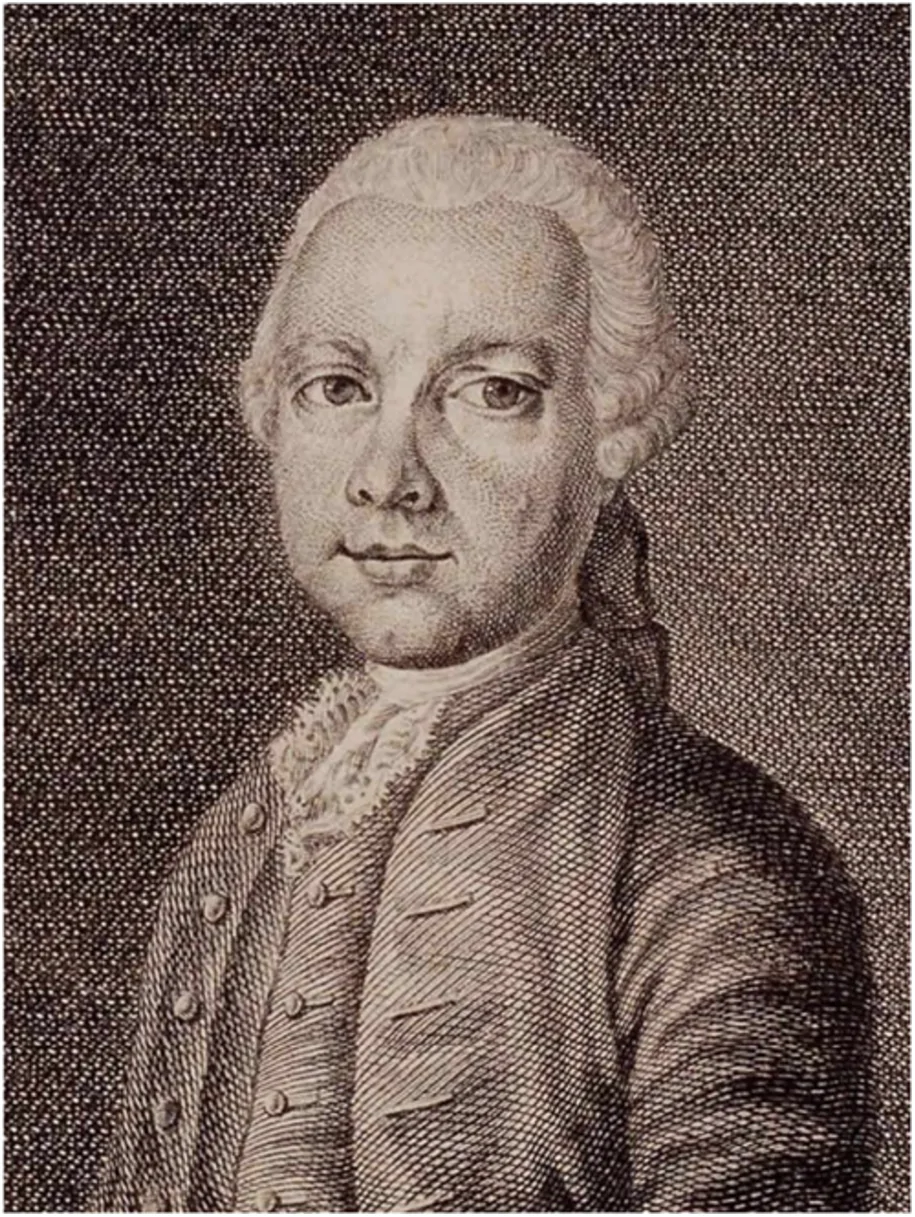
Portrait of Giovanni Antonio Scopoli (1723–1788).

Scopoli's *De hydrargyro Idriensi Tentamina physico‐chymico‐medica* [Physico‐Chemico‐Medical Essays on Idrija Mercury], published in Venice in 1761, is among the most detailed 18th‐century monographic accounts of occupational mercury poisoning in a specific mining community (Figure 2). Written during his long tenure as physician at the mercury mines of Idrija, at the time the second largest mercury mine in the world, the *Tentamina* combines mineralogical, chemical, and medical observations, culminating in a clinical discussion of diseases affecting mercury miners and smelter workers. Yet despite its historical significance, Scopoli's medical work remains largely unknown outside Central European historiography, overshadowed by his fame as a botanist and entomologist.

**Figure 2:**
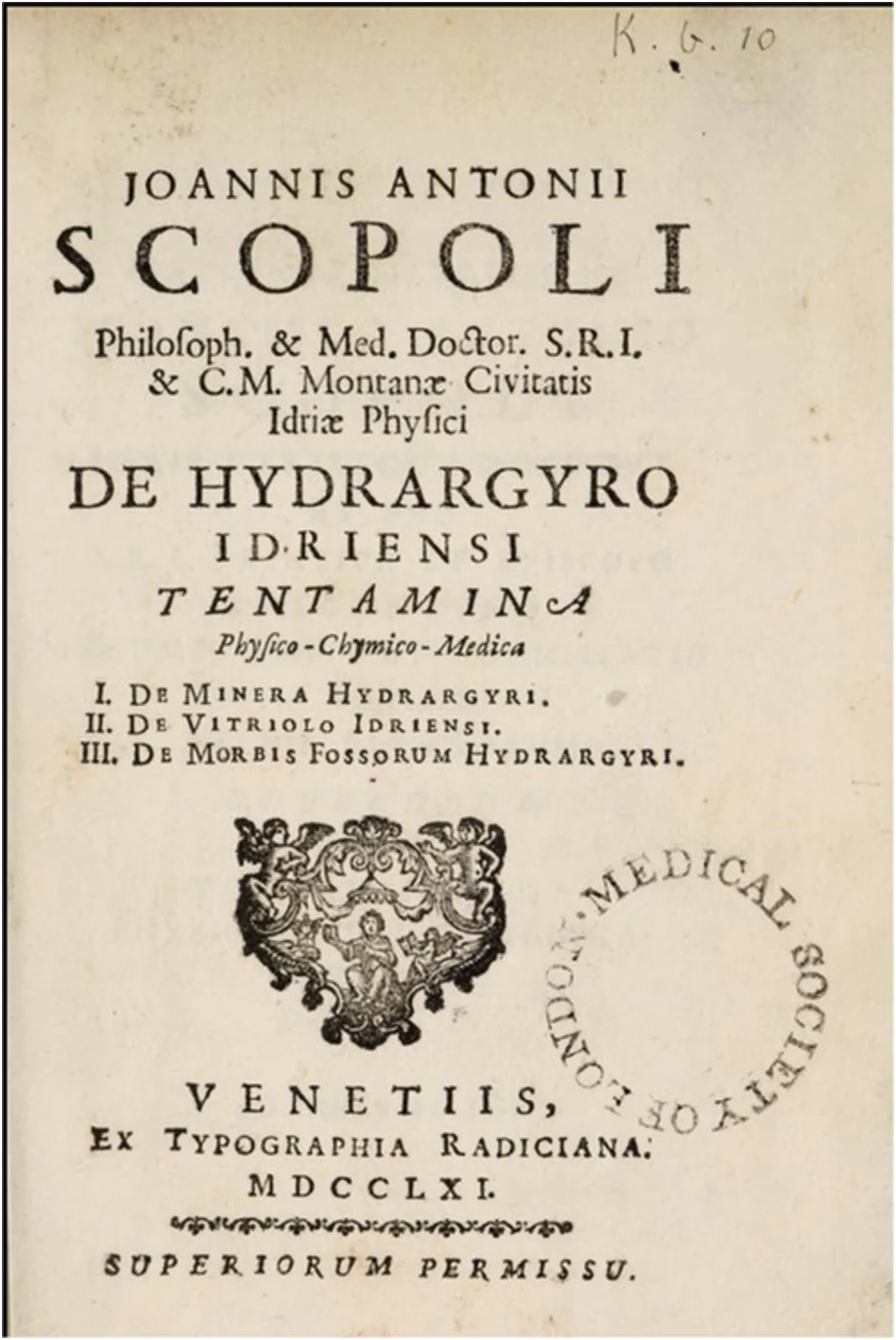
Frontispiece of the *De hydrargyro Idriensi tentamina physico‐chymico‐medica* [Physico‐Chemico‐Medical Essays on Idrija Mercury]. Venice; 1761.

This article reassesses the *Tentamina* as a significant but underexamined contribution to 18th‐century occupational medicine. We situate Scopoli's work within earlier writings on mining and mercury toxicity, compare his approach with Ramazzini's broader occupational nosology, and consider its relationship to later 19th‐century industrial medicine standards. The central argument is that Scopoli's text should not be read simply as a footnote to Ramazzini's work or as a premature version of modern toxicology, but as a distinct(ive) workplace‐based medical inquiry in which clinical observation, toxic exposure, workorganization, and, intriguingly, prevention, were brought into close relation. Scopoli did not found modern occupational medicine, nor did he possess the conceptual apparatus of 19th‐century toxicology. His importance lies in a different achievement: he produced a site‐specific, exposure‐specific, clinically grounded medical account of mercury work in which prevention followed directly from observation of the workplace conditions and of the workers' tasks.

The analysis draws on Scopoli's 1761 Latin text, with particular attention to the structure and arguments of the *De morbis fossorum hydrargyri*, and on 20th‐ and 21st‐century historical studies of medicine, mining, and occupational health in Idrija. Retrospective diagnostic terms such as mercurialism, silicosis, tuberculosis, or leptospirosis are used cautiously and analytically; they are not intended to imply that Scopoli possessed the pathological or microbiological concepts later associated with those conditions.

## 2 Historical Background: The Mercury Mine of Idrija

The mercury mine of Idrija, discovered by chance in 1490 when a local farmer noticed liquid mercury flowing into a stream, rapidly became one of the most strategically important industrial sites in the Habsburg Empire. By the sixteenth century, Idrija was producing mercury on an industrial scale, supplying the silver and gold mines of South America, where mercury was essential for the amalgamation process. At its peak, the Idrija mine was second in world production only to the Spanish mine of Almadén, and the two sites together effectively controlled the global mercury market for nearly four centuries [1].

The working conditions at Idrija were notoriously hazardous. Miners descended into shafts reaching depths of up to 200 m, working in poorly ventilated galleries filled with mercury vapor and cinnabar dust. The smelting process, which involved roasting cinnabar ore in furnaces to extract elemental mercury, exposed workers to acute and chronic mercury poisoning on a daily basis. The clinical consequences—tremor, salivation, neurological deterioration—were so prevalent and so disabling that they had been noted by observers well before Scopoli's arrival. Among the earliest was Paracelsus, who visited Idrija around 1521 and recorded his impressions in *Von der Französischen Krankheit* (1529) [2]:

Look at the example in Idrija: all who live there are lame or crippled, nearly suffocating or shivering, and will never be completely healthy again [3].

This passage is among the earliest recorded descriptions of mercurialism specifically associated with Idrija, and will be discussed further in the context of Scopoli's predecessors.

Medical and social care for miners had been introduced at Idrija earlier than at most comparable European sites, driven not by humanitarian concern but by economic necessity: chronic mercury poisoning severely reduced worker productivity, and the Habsburg administration had a direct financial interest in maintaining a functional workforce. A surgeon had been employed at the mine since the seventeenth century, and by 1740 a trained surgeon, Joseph Gutt, had been appointed with the specific mandate to prevent and treat professional mercury poisoning. In 1754, at the request of the mine director Anton Hauptmann, Giovanni Antonio Scopoli was appointed as the first fully qualified physician at Idrija, with free medical care extended to miners' families [4].

### 2.1 Giovanni Antonio Scopoli: Physician and Naturalist

Giovanni Antonio Scopoli was born on June 3, 1723, in Cavalese in the southern Tyrol, and died on May 8, 1788, in Pavia. He studied medicine at Innsbruck, graduating in 1753, and arrived at Idrija the following year. Scopoli remained at Idrija for 15 years, a period of considerable scientific productivity: it was here that he wrote the *Flora Carniolica* (1760) and the *Entomologia Carniolica* (1763), and maintained a celebrated correspondence with Carl Linnaeus (1707–1778). Scopoli's intellectual formation placed him firmly within the empiricist tradition of Enlightenment natural history. His correspondence with Linnaeus, which began in the late 1750s, was not merely an exchange of botanical specimens but a sustained scientific dialogue that shaped his approach to systematic observation and classification. Like Linnaeus, Scopoli was committed to the direct examination of natural phenomena in their specific environmental contexts, a habit of mind that informed not only his botanical and entomological work but also his medical practice at Idrija. His appointment as physician at the mine, secured through the patronage of the Habsburg administration at a moment when the economic importance of mercury production made the health of the workforce a matter of imperial concern, placed him in an unusually favorable position: a trained naturalist with systematic observational skills, stationed for an extended period at one of the most medically significant industrial sites in Europe. The botanical genus *Scopolia* and the alkaloid scopolamine bear his name. In 1769 he moved to Schemnitz (Banská Štiavnica), and in 1776 was appointed Professor of Botany and Chemistry at the University of Pavia.

Alongside these celebrated works in natural history, Scopoli produced the *Tentamina*, his principal occupational‐medical work, which has received comparatively little attention from historians of medicine outside Slovenia and Austria [5]. The work was published in Venice in 1761 and reprinted without the author's knowledge in Jena and Leipzig in 1771—a pirated edition that testifies to the text's circulation in German‐speaking scientific circles.

### 2.2 The *Tentamina*: Structure and Medical Content

The full title—*De hydrargyro Idriensi Tentamina physico‐chymico‐medica. I. De minera hydrargyri. II. De vitriolo Idriensi. III. De morbis fossorum hydrargyri*—announces the hybrid structure of the work: mineralogical, chemical, and medical. This structure is important for interpretation. Scopoli did not write a medical treatise detached from natural history or mining practice; rather, he placed the diseases of workers within a broader inquiry into the material, chemical, and environmental properties of the Idrija mercury complex. The first section, *De minera hydrargyri*, provides a detailed mineralogical description of the deposit. The second, *De vitriolo Idriensi*, examines the sulphurous components of the ore. The third and medically most significant section, *De morbis fossorum hydrargyri*, constitutes Scopoli's systematic clinical account of occupational mercury poisoning and related effects [6] (Figure 3).

**Figure 3:**
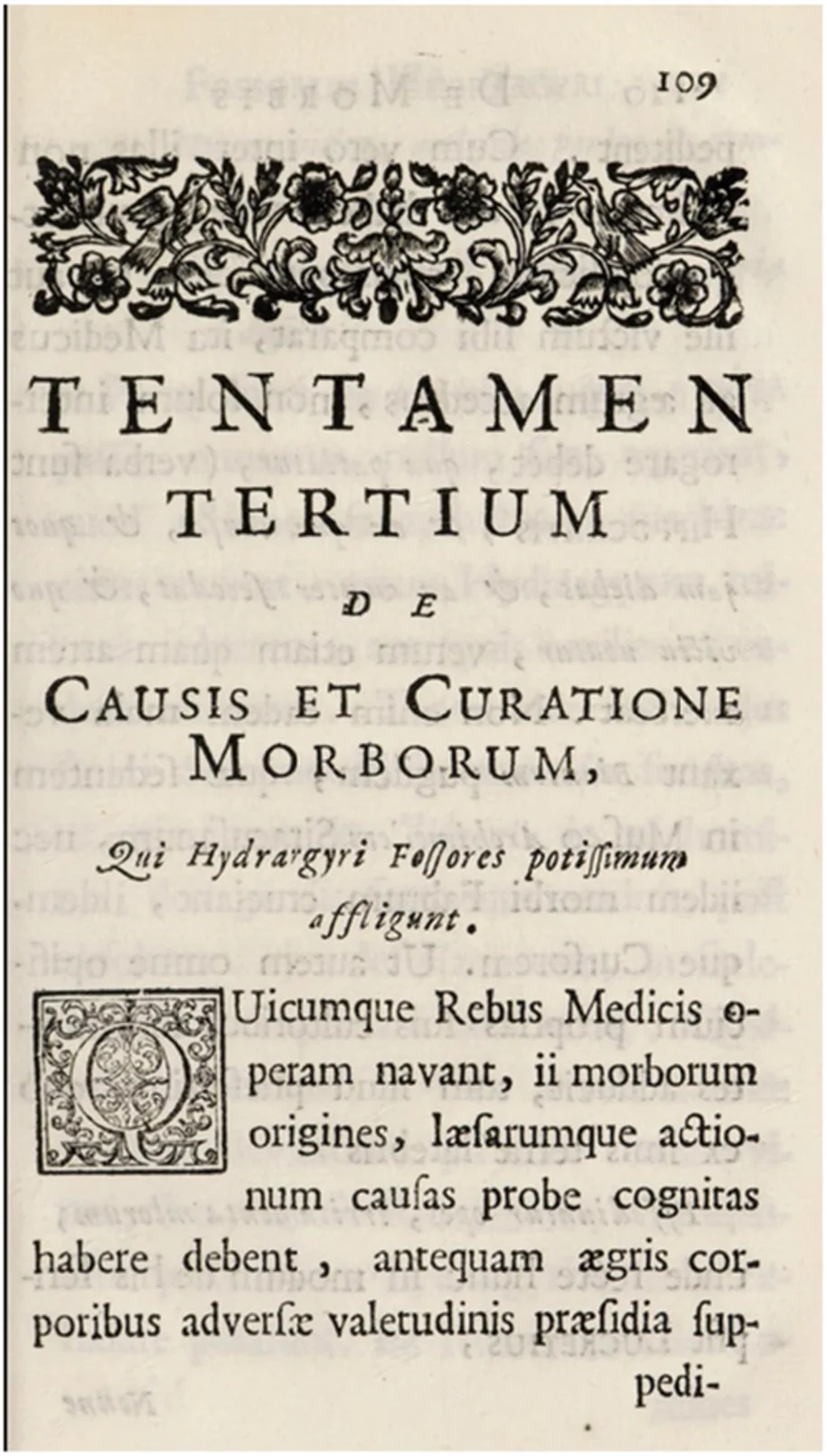
Start of the *Tentamen tertium: De causis et curatione morborum* [Third essay: On the causes and treatment of diseases], the third part devoted to the diseases of mercury miners (De Morbis Fossorum Hydrargyri.

### 2.3 Mercurialism: Clinical Description

In the medical section of the *Tentamina*, Scopoli's central concern is the occupational disease pattern associated with mercury exposure. He distinguishes two main clinical forms: a chronic form characterized by tremor, and a subacute form dominated by salivation and stomatitis. This differentiation, based on direct observation of miners and smelters over six years of practice at the time of writing (Scopoli himself specifies *per sexennium* in the text) though his total tenure at Idrija would extend to 15 years, reflects a sustained clinical engagement with mercury‐related disease that distinguishes the *Tentamina* from earlier, more fragmentary accounts [7].

Scopoli describes tremor as the most common and characteristic symptom among miners, accompanied by general weakness, fatigue, and loss of functional capacity. The clinical picture he draws is vivid and precise. He writes:

Mirum sane, adsidet fornacibus sanissimus homo, vel certis in locis intra Fodinas operatur, et subito tremulum, debilem…

[“It is indeed remarkable: a perfectly healthy man sits by the furnaces, or works in certain places inside the mines, and suddenly becomes tremulous and weak…”] [6, p. 114].

The causal attribution to mercury is stated with notable precision. In the Tremor Artuum section, Scopoli explicitly identifies mercury, and mercury alone, as the responsible agent:

Tale impedimentum ponit unicum, volatile factum hydrargyrum, humoribus nostris immixtum, absque ullo alterius causae satellitio

[“This impediment is produced solely by mercury rendered volatile, mixed into our bodily fluids, without the assistance of any other cause”] [6, p. 114].

He further specifies the mechanism of toxicity:

Solum ergo Hydrargyrum, in minimas particulas ab igne divulsum, et liquidis nostris immixtum, potens est superadditas musculorum vires ita frangere, ut morbus ille oriatur

[“Mercury alone, broken into the smallest particles by fire and mixed into our fluids, is capable of producing this disease”] [6, p. 114].

These passages leave no ambiguity: Scopoli does not attribute the tremor to mining work in general, but specifically and exclusively to inhaled mercury vapour.

The severity of the condition is conveyed with striking clinical detail. Scopoli records his direct observations of affected miners:

Vidi miseros, qui ambabus manibus firmiter arreptum vitrum aqua plenum priusquam labiis admovissent, excussisse penitus contentum liquidum, ut nec cibum, nec potum capere potuerint soli… Vidi quoque adeo debiles, ut propriis pedibus insistere cupientes, in terram protinus laberentur.

[“I have seen wretches who, having firmly grasped with both hands a glass full of water before bringing it to their lips, had entirely spilled its contents, so that they could take neither food nor drink unaided… I have also seen some so weak that, attempting to stand on their own feet, they fell immediately to the ground.”] [6, p. 116].

Scopoli identifies the quality of mine ventilation, proximity to furnaces, and duration of exposure as factors that influence the onset and severity of tremor. His recommended primary treatment is unambiguous in its causal logic:

Sola aeris liberioris usura saepe remedio est, multosque sine Medicina, hoc uno auxilio perductos vidi ad sanitatem.

[“The mere use of freer air is often sufficient remedy, and I have seen many restored to health by this one means alone, without medicine.”] [6, p. 120].

This therapeutic reasoning—that removal from the source of exposure is the primary and often sufficient intervention—reflects a clear understanding of the causal relationship between mercury vapor inhalation and neurological disease, and is one of the most clinically sophisticated aspects of the *Tentamina*.

The salivation syndrome is described as more prevalent among smelter workers than miners, a difference Scopoli attributes to the more intense and acute mercury vapor exposure at the furnaces. He traces the clinical progression from gingivitis and stomatitis:

foetor oris… tumor gingivarum, dentium praecipue molarium vacillatio, denudatio, nigredo

[“foul breath… swelling of the gums, loosening of the teeth especially the molars, their exposure and blackening”] [6, p. 128].

through tooth loss and ulceration to gangrenous involvement of the cheeks. His observation that tremor and stomatitis tend to present in different workers rather than in the same individual is stated with particular precision:

Qui salivatione laborant, nunquam aut raro tremuli apparent; magis autem illi, quibus dicta salivatio non contingerit.

[“Those who suffer from salivation never, or rarely, appear tremulous; while those in whom the said salivation does not occur are more commonly so.”] [6, p. 127].

Furthermore, Scopoli identifies the specific work task associated with salivation:

Malum hoc eos facile adoritur, qui tempore distillationis terreas retortas forte hiantes luto obducere solent.

[“This affliction easily attacks those who, during distillation, are accustomed to sealing with clay the earthen retorts that have cracked open.”] [6, p. 127].

From a modern perspective, these observations reflect an intuitive grasp of the relationship between exposure characteristics—route, intensity, duration, and specific task—and clinical manifestation, though Scopoli did not articulate it in those terms.

### 2.4 Beyond Mercurialism: Other Occupational Conditions

The *Tentamina* extends beyond mercury poisoning to describe a range of conditions that Scopoli associates with mining labor. Under the heading of “miners' asthma” (*asthma fossorum*), Scopoli describes a syndrome produced, in his account, by mineral dust and furnace fumes. Dust inhalation is identified as the primary cause of the associated cough:

Pulvis e lapidum fragmentis, eorumque acervis intra Fodinas undequaque diffusus

[“Dust from stone fragments and their accumulations, dispersed everywhere within the mines”] [6, p. 132].

The clinical progression he describes—persistent nocturnal cough, chest tightness, progressive dyspnea, and, in advanced cases, near‐suffocating respiratory failure—is compatible, in retrospect, with dust‐related pulmonary disease (pneumoconiosis), possibly complicated by tuberculosis, though neither condition was understood in pathological terms at the time [6, pp. 138–139, 8]. Scopoli supports his account with an autopsy observation of a 40‐year‐old smelter worker, whose lungs showed diffuse pleural adhesions and innumerable black lenticular spots throughout the pulmonary parenchyma, concluding:

En itaque mortem a pravis halitibus e minera hac exorta exhalantibus

[“Here, then, is a death caused by the noxious vapors exhaled from this ore”] [6, p. 120].

His preventive recommendations are practical and specific: miners should bind linen cloths over the mouth and nose when dust clouds cannot otherwise be contained, [6, p. 133] and smelter workers should cover their faces with glass masks—a proposal Scopoli traces to ancient practice recorded by Pliny, and advocates reviving for contemporary use [6, p. 140].

Scopoli also describes an intermittent fever among miners that, analyzed retrospectively, presents features more consistent with leptospirosis than with malaria. He introduces the condition with careful epidemiological specificity:

Nonnulla quoque annotanda sunt de febribus intermittentibus, quae homines nostros, vere, & autumno, saepissime invadunt

[“Some observations must also be recorded on the intermittent fevers, which very frequently attack our men in spring and autumn”] [6, p. 150]

He notes that the fevers worsen when spring water sources are turbid after heavy rainfall, are associated in severe autumnal cases with jaundice and frequently fatal dropsy, and respond inconsistently to Peruvian bark (quinine):

Cortex Peruvianus non eadem semper successus felicitate in hisce hominibus adhibetur

[“Peruvian bark is not always employed in these men with equal success”] [6, p. 153].

The absence of marshy ground near Idrija and the absence of enlarged spleen among affected miners—both noted by Scopoli—effectively argue against malaria, though Scopoli himself names no alternative etiology [7]. The quality of these clinical observations is characteristic of the *Tentamina* as a whole.

### 2.5 Social Medicine and Preventive Advocacy

The *Tentamina* engages explicitly with the social and economic conditions that shaped occupational disease at Idrija. Scopoli's position on working hours is stated with remarkable directness and corrects a widely circulated misconception. He writes:

Falsum etiam est illud, quod A. 1665 Societati Regiae Anglicanae relatum, & ejusdem Actis insertum fuit, Fossores nempe Idrienses ultra sex horas operari non posse; nam praeterquam quod unicae diei merces octo horarum laborem exigat, sunt etiam non pauci, qui pecuniae amore aut necessitate coacti diutius etiam operantur.

[“It is also false what was reported to the English Royal Society in 1665 and inserted in its Proceedings, namely that the miners of Idrija cannot work more than six hours; for beyond the fact that the wage of a single day requires 8 h of labor, there are also not a few who, out of desire for money or compelled by necessity, work even longer.”] [6, p. 112].

This passage is historically significant on multiple levels. It documents that the 6‐h limit had been recognized as a medical standard as early as 1665—cited as an established fact before the Royal Society—while simultaneously revealing that economic pressures had eroded this protection entirely by Scopoli's time. Scopoli thus identifies the gap between medically recommended exposure limits and actual practice as a direct consequence of wage structure and economic necessity—an argument that would not become standard in occupational medicine for more than a century.

He observes that miners suffering from tremor continued to descend into mercury‐laden galleries because their survival depended on their wages:

Sunt etiam non pauci, qui liberae pecuniae dura necessitate coacti, etsi debiles & tremuli, attamen Fodinas frequentare, suaque antra repetere, non desinunt, donec viribus exhausti domum transferantur.

[“There are also not a few who, compelled by the harsh necessity of money, although weak and tremulous, do not cease to frequent the mines and return to their galleries, until, exhausted of strength, they are carried home.”] [6, p. 121].

He explicitly addresses the mine administration, combining medical argument with economic reasoning:

Caveant itaque Rectores horum hominum, ne laesi jam & tremuli insalubribus Fodinae locis addicantur, vel sani etiam diutius, quam par est, ibidem relinquantur. Haec una regula observata potens est Operarios hosce a morbis praeservare, inutiles abolere sumptus, gentisque nostrae felicitatem diu ac multum promovere.

[“Let therefore the overseers of these men take care not to assign to the unhealthy places of the mine those who are already injured and tremulous, nor to keep even the healthy there longer than is appropriate. The observance of this single rule is capable of preserving these workers from disease, eliminating useless expenditure, and greatly and durably promoting the prosperity of our people.”] [6, p. 121].

This passage is among the most rhetorically forceful in the *Tentamina*. Scopoli appeals simultaneously to humanitarian concern, administrative responsibility, and economic self‐interest—a combination of arguments that reflects both the pragmatism of Enlightenment social medicine and the specific constraints of the Habsburg administrative context in which he was operating.

He further notes that the provision of free medication under imperial decree was of limited practical benefit:

…licet Augustae Dominae subditorum bonis perpetuo addicta liberalitas, miseris hisce gratis omnia, morborum curationibus necessaria, concesserit

[“…although the liberality of the August Sovereign, ever devoted to the welfare of her subjects, had granted to these wretches free of charge all that was necessary for the treatment of their diseases”] [6, p. 121].

Yet most miners could not afford to stop working regardless. These observations situate Scopoli within a tradition of socially engaged medicine in which the material conditions of labor are understood as determinants of disease, a perspective that would become more fully articulated in the reform medicine of the following century.

### 2.6 Methodological Character of the *Tentamina*

The methodological character of the *Tentamina* deserves attention in its own right, provided that the analysis remains grounded in what Scopoli actually did rather than in retrospective projections of later scientific categories onto an 18th‐century text.

What distinguishes the *Tentamina* from earlier writings on mercury toxicity is not the application of a formalized scientific method. Scopoli did not possess, and did not claim, the conceptual apparatus of modern epidemiology or experimental toxicology. Rather, it is the sustained and disciplined combination of several practices that, taken together, gave his clinical account its distinctive character: the confinement of inquiry to a specific and relatively stable workforce whose occupational exposures were known and directly observable; six continuous years of clinical practice at a single site; systematic attention to the conditions under which disease developed or was modified, both environmental and personal; and the consistent derivation of preventive recommendations directly from clinical and environmental observation.

This last feature—causal‐preventive reasoning grounded in workplace observation rather than in abstract medical theory—is perhaps the most distinctive aspect of the *Tentamina*. It is captured in the closing observation of the *De morbis fossorum hydrargyri*, where Scopoli, having completed his account of the diseases specific to mercury miners, draws an explicit boundary between occupational and general disease:

Hae sunt praecipuae aegritudines, quae Fossores Idrienses potissimum adfligunt, licet dentur & aliae, quae apud alias quoque gentes observantur.

[“These are the principal diseases that afflict the miners of Idrija above all, although there are also others that are observed among other peoples.”]

He then adds a clinically significant observation:

Id unum tamen consideratione dignum arbitror, quod apud nostros inflammatorii & acuti morbi longe rarius occurrant, quam aliis in locis.

[“I consider it worthy of note, however, that among our men inflammatory and acute diseases occur far less frequently than in other places.”] [6, pp. 159–160].

These features do not make Scopoli a proto‐epidemiologist or a founder of industrial toxicology. They make him a careful and sustained clinical observer who worked within the empiricist tradition of Enlightenment natural history and applied its methods—careful description, attention to environmental context, reasoning from observation to practical consequence—to the specific conditions of mercury mining at Idrija.

### 2.7 Reception and Legacy

The *Tentamina* was not without immediate impact. The unauthorized 1771 reprint in Jena and Leipzig produced by J.C.T. Schlegel testifies to the text's circulation in German‐speaking scientific circles. It should be noted, however, that the German edition omitted the section on miners' diseases, reproducing only the mineralogical and chemical portions of the work; the medical content was thus less widely disseminated in translation than the natural history sections [7].

The most comprehensive scholarly analysis of the *Tentamina* in the twentieth century was provided by the Viennese historian of medicine Erna Lesky, whose monograph *Arbeitsmedizin im 18. Jahrhundert: Werksarzt und Arbeiter im Quecksilberbergwerk Idria* (1956) situated Scopoli's work within the broader context of Enlightenment social medicine and Habsburg administrative reform [5]. The Slovenian medical historian Ivan Pintar had earlier published a detailed analysis of the medical content of the *Tentamina* in 1954 [7], and the Croatian historian Lavoslav Glesinger contributed important contextual studies on mercurialism in the Adriatic region [9]. More recently, Zvonka Zupanič Slavec has examined Scopoli's work within the history of Slovenian occupational medicine, emphasizing its significance as the earliest systematic clinical study of mercury poisoning in the Idrija context [10].

Despite this body of Central European scholarship, the *Tentamina* remains substantially underexamined in Anglophone histories of occupational medicine. George Rosen's *The History of Miners' Diseases* (1943), which traces the development of knowledge about occupational lung disease from antiquity to the twentieth century, mentions Scopoli only in passing, noting that “in 1761, an Italian, Giovanni Scopoli, described mercury poisoning, with the characteristic trembling, among the miners around Alto Isonzo.“ [11] The *Tentamina* itself is not cited, and no analysis of its clinical content, methodological character, or social medicine dimension is offered. Rosen's cursory treatment is representative of the broader Anglophone tendency to acknowledge Scopoli's name while leaving his work unexamined. More recent Anglophone scholarship on the history of industrial medicine and toxicology has similarly tended to locate the discipline's origins in the Industrial Revolution and its aftermath, leaving the 18th‐century European tradition of site‐specific occupational medicine underexplored [12]. The present article is intended as a contribution toward addressing that gap.

## 3 Discussion

The evidence reviewed here supports a more precise placement of Scopoli within the history of occupational medicine. The *Tentamina* should be understood neither as a regional curiosity nor as a displaced foundation of the modern discipline, but as a significant 18th‐century monograph on occupational mercury disease that occupies a distinctive position between the broad nosological survey of Ramazzini and the reform‐oriented clinical medicine of the early 19th century.

Scopoli's work belonged to a long European tradition of writing on mining hazards, toxic vapors, and mercury‐related disease. The toxicity of mercury had been recognized since antiquity: Pliny and Dioscorides described acute poisoning, and medieval Arabic physicians were familiar with the syndrome. The systematic study of occupational mercury exposure, however, developed from the sixteenth century onward in the mining regions of Central Europe.

Ulrich Ellenbog's *Von den gifftigen besen Tempffen und Reuchen* (written 1473, printed 1524) warned craftsmen, primarily goldsmiths, about the dangers of toxic vapors in the workplace. Georg Bauer Agricola's *De re metallica* (1556) described mining hazards systematically, including mercury vapor exposure. Paracelsus, whose visit to Idrija around 1521 was noted above, developed his observations on mercurialism more fully in *Von der Bergsucht und anderen Bergkrankheiten* (1533–34), describing tremor, salivation, dental disease, and renal involvement, and proposing a rudimentary respiratory device for the protection of miners [3]. Pierandrea Mattioli (1500–1577), who practiced for fourteen years in nearby Gorizia, published observations on chronic mercury poisoning in 1554 [13]. Scopoli was familiar with this tradition and positioned his own work in relation to it. What distinguishes the *Tentamina* from these predecessors is not the recognition of mercury toxicity as such, that had been established by Paracelsus and others, but a combination of features that none of them achieved individually or collectively. First, duration: where Paracelsus visited Idrija on a single occasion and Mattioli practiced in the nearby region of Gorizia, Scopoli's account is the product of six continuous years of clinical observation at the same site, among the same workforce. Second, clinical depth: the *Tentamina* moves beyond symptom enumeration to include individual case histories, anatomopathological observation (the autopsy of the 40‐year‐old smelter worker), instrumental measurement (thermometry), and systematic comparison of clinical presentations across different worker groups. Third, causal integration: where Agricola and Paracelsus describe occupational hazards and propose remedies in relative isolation from one another, Scopoli constructs an explicit chain connecting type of exposure, route of absorption, clinical manifestation, and specific preventive measure. Fourth, social analysis: the *Tentamina* engages with wage structure, working hours, and the gap between medically recommended exposure limits and actual practice, dimensions entirely absent from the earlier mining literature. It is the combination of these elements, rather than any single innovation, that gives the *Tentamina* its distinctive character within the pre‐Ramazzinian and post‐Ramazzinian traditions alike.

The relationship between the *Tentamina* and Ramazzini's *De morbis artificum diatriba* (Modena, 1700) is best understood as one of complementarity rather than supersession. Ramazzini offered a broad comparative nosology across many occupations, including workers exposed to mercury, such as gilders and mirror‐makers, and added to the Hippocratic anamnesis the celebrated question: “What occupation does the patient follow?“ [14] Where Ramazzini's treatment of mercury‐exposed workers is necessarily brief, a consequence of the encyclopedic scope of his project, Scopoli's account is grounded in continuous on‐site clinical practice among several hundred miners and smelters, producing a level of clinical and causal specificity that Ramazzini's method could not achieve [15]. Scopoli's preventive and social recommendations also go further: while Ramazzini described occupational diseases with considerable acuity, the *Tentamina* gives greater prominence to site‐specific preventive measures and to the administrative problem of protecting sick workers from continued exposure.

The parallels with 19th‐century successors are instructive without being overstated. Charles Turner Thackrah (1795–1833), a Leeds surgeon whose *The Effects of Arts, Trades, and Professions, and of Civic States and Habits of Living, on Health and Longevity* (1831) is widely regarded as the founding text of British occupational medicine [16], shared with Scopoli a commitment to direct workplace observation, systematic clinical documentation, and explicit social criticism. Both physicians identified the reduction of working hours and the introduction of protective measures as primary preventive strategies, and both argued that occupational diseases were not inevitable consequences of industrial labor but products of modifiable conditions. The social and political environments in which they operated differed considerably—Thackrah within the context of early industrial capitalism and its reform movements [17], Scopoli within the Habsburg administrative framework—but the structural parallel between their approaches remains historically significant. The French physician Louis Tanquerel des Planches (1810–1862), whose *Traité des maladies du plomb ou saturnines* (1839) remains the definitive 19th‐century clinical study of occupational lead poisoning, provides a further point of comparison: his methodology of systematic case series, detailed symptom classification, and correlation of clinical findings with occupational exposure history is precisely the approach that Scopoli had applied to mercury poisoning in a single industrial community nearly eighty years earlier [18].

Several features of the *Tentamina* merit particular emphasis in this reassessment. Its monographic depth distinguishes it from all previous writing on occupational mercury disease. Where Ramazzini surveyed many trades across a broad canvas, Scopoli focused all his analytical resources on a single toxic substance in a single industrial community over an extended period. This monographic approach—later exemplified by Tanquerel des Planches on lead and by the 19th‐century literature on specific industrial poisons—produced a level of clinical specificity and etiological coherence that broader surveys could not achieve.

Its clinical originality is evident in several specific contributions: the explicit identification of mercury as the sole causal agent of tremor, stated with notable precision; the differentiation of chronic neurological from subacute mucosal mercurialism on the basis of exposure type and intensity; the retrospectively plausible description of dust‐related pulmonary disease supported by autopsy evidence; and the clinical account of intermittent fever that, in retrospect, is more consistent with leptospirosis than with any other condition known to have been endemic at Idrija.

Its social medicine dimension places Scopoli in a tradition of medically informed social criticism that would become fully articulated only in the following century. His analysis of the relationship between low wages, compulsory overtime, and occupational disease; his documentation of the gap between medically recommended exposure limits and actual practice; and his direct address to the mine administration combining humanitarian, medical, and economic arguments, all anticipate the reform orientation that would characterize Thackrah, Villermé, and the industrial medicine of the 19th century.

Its preventive orientation—the consistent derivation of practical preventive measures from clinical and environmental observation—gives the *Tentamina* its most distinctive character as an occupational‐medical text. Scopoli's proposals for respiratory protection, workplace ventilation, reduced exposure time, and removal of symptomatic workers from hazardous environments constitute an early and coherent framework for occupational health intervention, grounded in direct observation of the workplace rather than in theoretical medicine.

The continuing relevance of these themes should not go unnoticed. Mercury remains a significant occupational and environmental health concern: the Minamata Convention on Mercury (2013), ratified by over 140 countries, reflects the persistence of mercury toxicity as a global public health problem, particularly in artisanal and small‐scale gold mining communities where exposure conditions bear some resemblance to those Scopoli described at Idrija more than two and a half centuries ago [19]. The history of how physicians first learned to observe, describe, and propose remedies for mercury poisoning in an occupational setting retains more than antiquarian interest.

## 4 Conclusions

Giovanni Antonio Scopoli's *De hydrargyro Idriensi Tentamina physico‐chymico‐medica* (1761) is a significant and underexamined contribution to the history of occupational medicine. Based on six years of direct clinical observation at one of the world's principal mercury mining sites, it offers a systematic, clinically detailed, and socially engaged account of occupational mercury poisoning and related conditions. Its methodological character—site‐specific, exposure‐specific, clinically grounded, and consistently oriented toward prevention—places it at a distinctive point in the development of occupational medicine as a form of medical inquiry.

Situated between Ramazzini's encyclopedic nosology and the reform‐oriented empiricism of Thackrah and his contemporaries, the *Tentamina* represents neither a founding text nor a premature version of modern industrial toxicology, but a substantial achievement of 18th‐century occupational‐medical observation. Scopoli's engagement with the clinical, environmental, and social dimensions of mercury work at Idrija produced a monograph whose depth and coherence have not been adequately recognized outside Central European medical historiography.

The *Tentamina* deserves a more prominent place in Anglophone histories of occupational medicine—not as a founding text, but as a significant example of the depth and variety of pre‐industrial occupational medical inquiry, and as evidence that the intellectual foundations of the discipline were laid well before the Industrial Revolution transformed the conditions of labor in Europe.

## Author Contributions

Conceptualization: Alberto Zanatta, Paola Mason, Sofia Bollini. Investigation and historical research: Alberto Zanatta, Paola Mason, Sofia Bollini. Writing – original draft: Alberto Zanatta, Paola Mason, Sofia Bollini. Writing – review and editing: Alberto Zanatta, Paola Mason, Sofia Bollini, Giovanni Magno. Supervision: Alberto Zanatta.

## Funding

The authors have nothing to report.

## Conflicts of Interest

The authors declare no conflicts of interest.

## Institutional Review Board Statement/Ethics Approval and Informed Consent

Ethics approval was not required for this study because it is based exclusively on historical published sources and does not involve human participants, human material, personal data, or animal subjects.

## Data Availability

The data that support the findings of this study are available from the corresponding author upon reasonable request.
